# Genome-Wide Identification of the Potato GGPS Gene Family and Analysis of Its Response to Abiotic Stress

**DOI:** 10.3390/genes16060646

**Published:** 2025-05-28

**Authors:** Changqing Fu, Wei Li, Xiaotian Chen, Shunjuan Gao, Mingfei Jia, Shuqing Zhang, Jianghui Cui

**Affiliations:** 1 College of Agronomy, Hebei Agticultural University, Baoding 071000, China; 17863337648@163.com (C.F.); liwei57350@163.com (W.L.); 16607924109@163.com (S.G.); 2 Hebei Key Laboratory of Crop Germplasm Resources, Hebei Agticultural University, Baoding 071000, China; 3 North China Key Laboratory for Crop Germplasm Resources of Education Ministry, Hebei Agticultural University, Baoding 071000, China; 4 Weichang County Manchu and Mongolian Autonomous County Potato Research Institute, Chengde 067000, China; xiaotian0301@163.com; 5 Shijiazhuang Academy of Agriculture and Forestry Sciences, Shijiazhuang 050080, China; 15733223865@163.com (M.J.); sjzzsq@126.com (S.Z.)

**Keywords:** potato, GGPS gene family, carotenoid synthesis, abiotic stress, gene expression analysis

## Abstract

Background: Geranylgeranyl pyrophosphate synthase (GGPS) is a pivotal enzyme in terpene biosynthesis, influencing the production of carotenoids, chlorophylls, and diverse phytohormones. This study aimed to identify and characterize the *StGGPS* gene family in potato (*Solanum tuberosum*) to elucidate its involvement in carotenoid synthesis and responses to abiotic stresses. Methods: Employing bioinformatics approaches, including HMMER, SMART, and Pfam, we conducted a genome-wide identification of StGGPS genes. Subsequent phylogenetic analysis, gene structure characterization, conserved motif detection, and synteny analysis were performed to investigate evolutionary relationships within the family. The expression patterns of StGGPS genes were then analyzed using RNA-seq data and quantitative real-time PCR (qRT-PCR) in potato tubers exhibiting different pigmentation and under drought and salt stress conditions. Results: Eleven *StGGPS* genes were identified, unevenly distributed across seven chromosomes, and classified into three subfamilies based on phylogenetic and structural analyses. Synteny analysis revealed one intra-genomic duplicate pair (*StGGPS1*/*StGGPS4*) and conserved orthologs with other Solanaceae species. Promoter analysis identified cis-elements related to light response and abiotic stress (e.g., ABRE and CGTCA-motif). Expression data showed differential regulation of StGGPS genes in colored tubers, with yellow and red tubers exhibiting higher expression of carotenoid-related genes. Under drought stress, *StGGPS10* was significantly upregulated (5.2-fold, *p* < 0.001), while *StGGPS6* showed salt-responsive induction (3.8-fold, *p* < 0.001), linking them to ABA signaling and cytoskeletal dynamics, respectively. Conclusions: This study provides a comprehensive overview of the StGGPS gene family, highlighting their roles in carotenoid biosynthesis and abiotic stress responses. The stress-specific expression patterns of *StGGPS10* and *StGGPS6* offer potential targets for genetic improvement of potato stress resilience.

## 1. Introduction

Carotenoids, a diverse class of terpenoid pigments, are ubiquitous in bacteria, fungi, algae, and terrestrial plants, where they fulfill critical functions in photosynthesis and photoprotection [1,2,3,4,5,6]. Within photosynthetic membranes, carotenoids associate with proteins to form pigment–protein complexes, acting as integral light-harvesting components [7]. Beyond their direct roles in photosynthesis, carotenoids contribute to plant development and stress resistance by serving as precursors for retrograde signaling molecules [8]. For example, carotenoids are metabolized via the methylerythritol phosphate (MEP) pathway to synthesize abscisic acid [9], a phytohormone that enhances plant resilience to abiotic stresses such as drought and salinity [10]. Furthermore, strigolactones, another group of carotenoid-derived plant hormones, regulate plant architecture by inhibiting shoot branching, modulating root development, and promoting leaf expansion, thereby influencing plant aging and secondary growth processes [11].

Geranylgeranyl pyrophosphate synthase (GGPS) is a pivotal enzyme in terpenoid biosynthesis, catalyzing the formation of geranylgeranyl pyrophosphate (GGPP) from farnesyl pyrophosphate (FPP) and isopentenyl pyrophosphate (IPP) [12]. GGPP functions as a precursor for the synthesis of crucial plant pigments and hormones, including carotenoids, chlorophyll, gibberellin, and abscisic acid [13]. Accumulating evidence from diverse plant species underscores the multifaceted role of GGPS in both primary metabolism and stress adaptation. For instance, in sweet potato (*Ipomoea batatas*), *IbGGPS* overexpression enhances osmotic stress tolerance by modulating carotenoid biosynthesis [14], while, in cotton (*Gossypium hirsutum*), GGPS isoforms regulate chlorophyll synthesis under abiotic stress [15]. Analogously, Liriodendron tulipifera GGPS controls terpenoid accumulation with tissue-specific expression patterns [16], and Arabidopsis *AtGGPS11* is essential for photosynthesis-related isoprenoid production [17]. Taken together, these findings suggest that GGPS gene families exhibit functional diversification, likely shaped by evolutionary pressures to fulfill species-specific metabolic and environmental requirements.

Potato (*S. tuberosum*), the most productive noncereal food crop, is a vital source of carbohydrates, dietary fiber, vitamins, and antioxidants [18]. Despite its nutritional importance, potato cultivation is highly vulnerable to abiotic stresses such as drought, salinity, and heat [19]. Although GGPS-mediated terpenoid metabolism is recognized as a critical component of stress resilience in other plants [14,15,16,17], the functional roles of the potato GGPS gene family remain unexplored. This study was guided by the hypothesis that the potato GGPS family has evolved functional divergence to regulate carotenoid biosynthesis and abiotic stress adaptation, with distinct members contributing to stress-specific responses. 

To investigate this hypothesis, we conducted a comprehensive analysis of the GGPS family in the potato genome, encompassing characterization of physicochemical properties, phylogenetic relationships, and subcellular localization. Furthermore, we explored the evolutionary mechanisms underlying gene family expansion, including duplication events and collinearity analyses with homologs in model species such as Arabidopsis, tomato, and pepper. Finally, we validated the functional association of candidate genes with abiotic stress responses through transcriptomic profiling and quantitative PCR, with a specific focus on drought and salt stress due to their significant impact on potato productivity. By integrating evolutionary insights with expression dynamics, this study elucidates the role of functional specialization within the *StGGPS* gene family in contributing to stress resilience and identifies promising targets, such as *StGGPS6* and *StGGPS10*, for breeding programs aimed at developing stress-tolerant cultivars.

## 2. Materials and Methods

### 2.1. RNA Extraction and qRT-PCR Analysis

The potato variety Jinong Shu 8511, bred by the Agricultural University of Hebei, served as the experimental material for this study. The plants, characterized by yellow skin and flesh, were grown for 21 days before being subjected to stress conditions. To simulate drought stress, seedlings were treated with 25 mmol/L PEG6000 [18] and, to simulate salt stress, 70 mmol/L of NaCl was used [20]. Samples were collected at 0, 12, and 24 h post-stress, flash-frozen in liquid nitrogen, and stored at −80 °C.

Total RNA was extracted using the Promega RNA Extraction kit (LS1040, Promega Corporation, Madison, WI, USA), and RNA integrity and concentration were assessed through agarose gel electrophoresis and a Nanodrop ND-2000 spectrophotometer (Nanodrop Technologies, now part of Thermo Fisher Scientific, Waltham, MA, USA). First-strand cDNA synthesis was performed using the Kangwei Century RT gDNA kit (CW2020M, Kangwei Century Biotechnology Co., Ltd., Beijing, China). qRT-PCR was carried out on a CFX96 system (Bio-Rad Laboratories, Inc., Hercules, CA, USA) using the US EVERBRIGHT AugeGreen qPCR Master Mix (S2008L, US EVERBRIGHT Biotechnology, Santa Clara, CA, USA) The reaction conditions were based on the AugeGreen qRT-PCR Master Mix instructions. qRT-PCR assays were performed with three biological replicates and three technical replicates. Significant differences were determined by one-way ANOVA using SPSS 26.0. StEF-1α was used as the reference gene [21], and relative expression was calculated using the 2^−ΔΔct^ methods [22].

### 2.2. Identification and Sequence Analysis of Potato GGPS Genes

The genome annotation files and nucleotide sequences for *S. tuberosum* were downloaded from the Ensembl Plant database (http://plants.ensembl.org/index.html, on 1 May 2024). The GGPS structural domain (PF00348) was identified using HMMER 3.1 (http://hmmer.org/download.html, on 5 May 2024) within the potato genome protein sequences. Sequences lacking the GGPS structural domain were excluded, and the remaining sequences were verified using SMART (http://smart.embl-heidelberg.de, on 11 May 2024) and Pfam (v.36, https://www.ebi.ac.uk/interpro/, on 11 May 2024) tools. The physical and chemical properties of the identified GGPS family members, including amino acid count, molecular weight, and theoretical isoelectric points, were analyzed using BioPerl (v1.5.1) [23].

### 2.3. Phylogenetic Analysis

Multiple sequence alignments of potato GGPS, along with GGPS sequences from Arabidopsis, tobacco, tomato (SlGGPS), and pepper, were performed using the MUSCLE algorithm. A phylogenetic tree was constructed using IQ-TREE, with 1000 bootstrap replicates [24].

### 2.4. Gene Structure, Conserved Motifs, and 3D Structural Analysis

Gene structure information for potato GGPS family members was extracted from genome annotation files, and intron/exon structures were visualized using GSDS 2.0 (http://gsds.gao-lab.org/, on 25 May 2024) [25]. Conserved motifs were identified using the MEME 5.5.5 online tool (http://meme-suite.org/, on 25 May 2024) with the following parameters: a maximum number of 10 motifs, a motif width of 6–50, amino acids, and default settings for other parameters [26]. Subcellular location predictions were conducted using WoLF PSORT [27]. The three-dimensional structural models of potato GGPS genes were retrieved from the RCSB PDB database (https://www.rcsb.org/, on 27 May 2024) and refined using PSI-BLAST, SWISS-MODEL (https://swissmodel.expasy.org/, on 25 May 2024), and SAVES (https://saves.mbi.ucla.edu/, on 25 May 2024) for quality evaluation [28,29].

### 2.5. Analysis of Cis-Acting Elements in Promoter Regions

The cis-acting elements within 2 kb upstream of the potato GGPS gene promoters [30] were analyzed using the PlantCARE database, and the elements were visualized using the GGPS 2.0 website (http://gsds.gao-lab.org/, on 27 May 2024) [25].

### 2.6. Chromosomal Location, Gene Distribution, and Synteny Analysis

The chromosomal locations of *StGGPS* family members were extracted from genome annotation files and mapped using TBtools II (TBtools v1.120) [31]. Duplication events among potato GGPS genes were analyzed with MscanX (MscanX v1.0) software [32], and synteny relationships were visualized using Circos (Circos v0.69-14) [33]. Synonymous substitution (Ks) and nonsynonymous substitution (Ka) rates for duplicated gene pairs were calculated using KaKs Calculator 2.0 [34]. Furthermore, MScanX software was used to analyze the collinearity between potato GGPS genes and multiple species.

### 2.7. Expression Analysis of RNA Data

RNA-seq raw counts were normalized using the DESeq2 package (v1.30.1) with median-of-ratios method. RNA-seq data were obtained from NCBI to assess the expression pattern of GGPS genes in potato tubers of various colors (white, yellow, red, and purple) and under abiotic stresses, including salt stress (200 mM NaCl for 12 and 24 h) and simulated drought stress (150 mM mannitol for 12 and 24 h). A heat map of gene expression was generated using TBtools II [31].

## 3. Results

### 3.1. Identification of GGPS Gene Family Members and Analysis of Protein Physicochemical Properties in Potatoes 

Eleven GGPS (*StGGPS*) gene family members were identified in potatoes using HMMER 3.1 in conjunction with the SMART, Pfam, and NCBI CDD databases. The *StGGPS* genes were sequentially named (*StGGPS1*–*StGGPS11*). BioPerl analysis showed that the amino acid length of *StGGPS* family members ranges from 294 to 398 residues, with an average length of 351 residues. The molecular weight varies from 32,784.6 to 43,490.4 Da, with an average molecular weight of 38,973.8 Da. The theoretical isoelectric point ranges from 4.87 to 8.38, with an average of 6.41. Among the *StGGPS* genes, six exhibit predominant localization in the chloroplast. Two genes show subcellular distribution across the cytoplasm, nucleus, and mitochondria, while one gene is associated with the cytoskeleton (Table 1). 

### 3.2. Potato GGPS Family Evolution Analysis, Gene Structure, and Conserved Motifs

**Figure 1:**
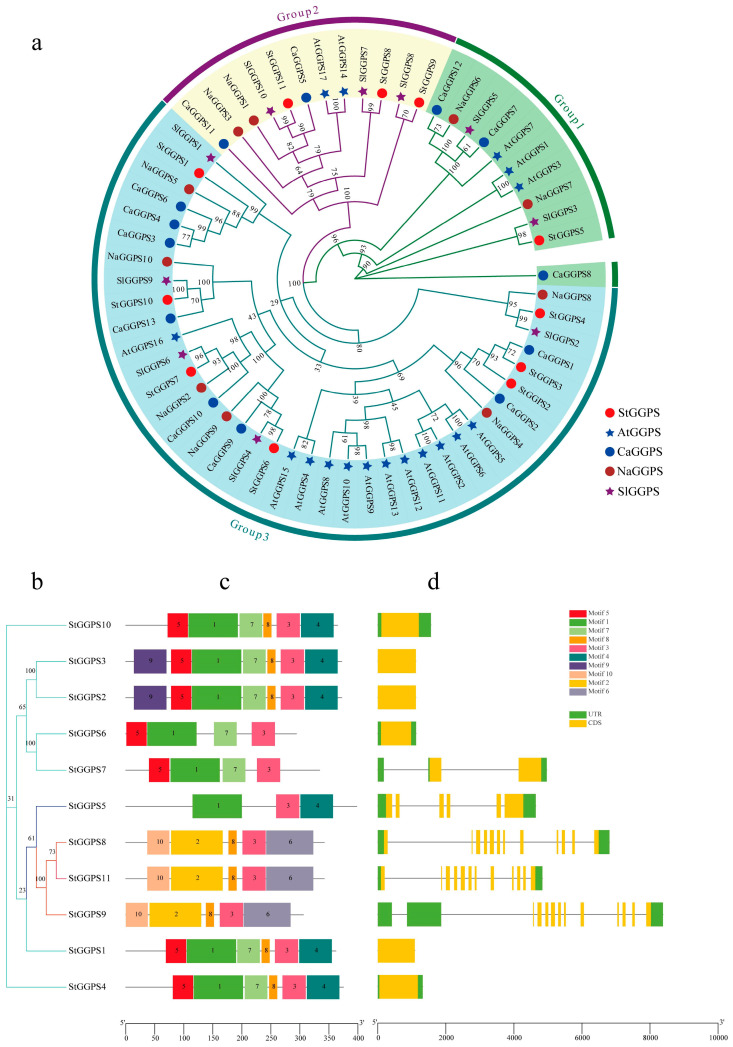
Phylogenetic, gene structure, and conserved motif analysis of the *GGPS* gene family. (**a**): Phylogenetic tree of the GGPS gene family in *A. thaliana*, *S. tuberosum, N. attenuata*, *S. lycopersicum*, and *C. annuum.* (**b**): Phylogenetic tree of the *StGGPS* gene family. (**c**): Conserved motif analysis of the *StGGPS* gene. (**d**): Analysis of *StGGPS* gene structure.

To explore the evolution of the GGPS gene family, a phylogenic tree was constructed using genes from Arabidopsis (*Arabidopsis thaliana*), potato (*S. tuberosum*), tobacco (*Nicotiana attenuata*), tomato (*Solanum lycopersicum*), and hot pepper (*Capsicum annuum*) (Figure 1). The GGPS genes were divided into three subfamilies: Group 3, with seven potato *StGGPS* members; Group 2, with three potato *StGGPS* members; and Group 1, with one potato *StGGPS* member. Members within the same branch likely have similar functions and evolutionary relationships.

**Figure 2:**
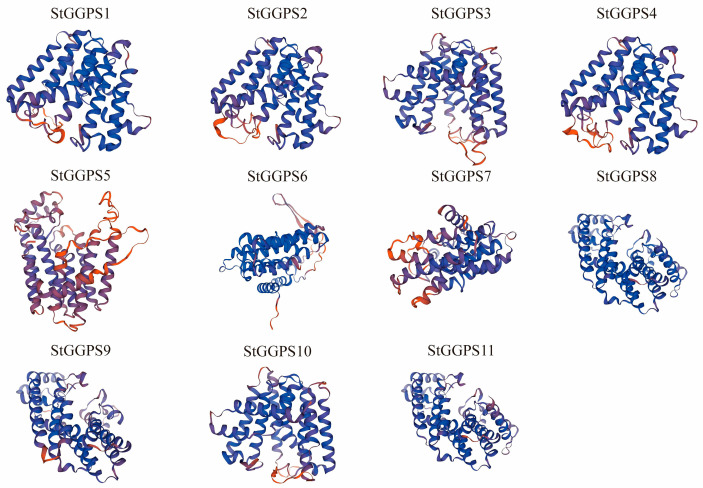
Three-dimensional structural modeling of proteins of the *StGGPS* gene family.

To further elucidate the evolutionary relationships among *StGGPS* genes, we analyzed the intron–exon substructure and conserved motifs of potato *StGGPS* members (Figure 1). The genetic structure of different *StGGPS* members exhibits considerable variation, ranging from intronless genes to those containing up to 11 introns. This diversity in gene structure suggests that *StGGPS* genes may have been subjected to divergent selection pressures during evolution. Notably, *StGGPS* genes within the same subfamily generally displayed similar gene structures, a pattern consistent with the phylogenetic tree, indicating a strong correlation between the intron–exon gene structure of *StGGPS* genes and their phylogenetic relationships. Given the association of conserved sequence elements with protein function, we employed MEME to identify 10 distinct motifs (Motif 1 through Motif 10). The number of motifs present in each *StGGPS* member ranged from three to seven; for instance, *StGGPS5* contains a minimum of three motifs, while *StGGPS2* and *StGGPS3* possess a maximum of seven. Although certain motifs are absent in some *StGGPS* members, a conserved pattern, particularly Motif 3, is consistently observed across all *StGGPS* genes. Furthermore, analysis of the predicted three-dimensional structures revealed a higher degree of structural similarity among proteins within the same subfamily compared to those from different subfamilies. For example, *StGGPS8*, *StGGPS9*, and *StGGPS11* from Group 2 exhibit similar structures, as do *StGGPS1*, *StGGPS2*, *StGGPS3*, *StGGPS4*, and *StGGPS10* from Group 3 (Figure 2). Collectively, this integrated analysis of the phylogenetic tree, gene structure, and conserved motifs suggests that *StGGPS* members within the same subfamily share similar gene functions and protein structures, implying analogous functional roles. We hypothesize that the observed similarities in gene structures and conserved motifs among *StGGPS* genes are indicative of functional convergence.

### 3.3. Chromosome Localization of Genes and Analysis of Gene Duplication Events 

**Figure 3:**
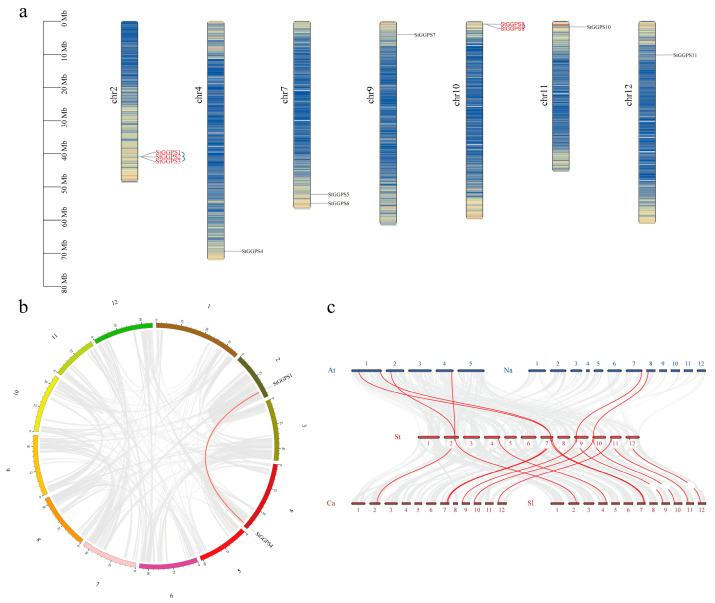
Gene duplication events in the *GGPS* family of *S. tuberosum.* (**a**): Chromosomal localization map of *StGGPS* gene. (**b**): Covariance analysis of *StGGPS* gene. (**c**): Collinearity analysis of GGPS genes in multiple species.

Chromosomal positions of *StGGPS* family members were extracted from the genome annotation file using Tbtools II. We found that chromosome 2 contained three *StGGPS* genes; chromosomes 7 and 10 contained two *StGGPS* genes; and chromosomes 4, 9, 11, and 12 contained only one *StGGPS* gene (Figure 3a).

To explore the structural and functional characteristics of the *StGGPS* genes, we examined gene duplications through tandem repeats and segmental duplications. We identified *StGGPS* gene pairs resulting from duplication events, specifically *StGGPS1*/*StGGPS4* (Figure 3b). We found three pairs of tandem repeat genes: *StGGPS1*, *StGGPS2*, *StGGPS2*, *StGGPS3*, *StGGPS8*, and *StGGPS9*. Gene duplication and fragment duplication likely drove the evolution of *StGGPS* genes. Further analysis of the Ka/Ks ratios for each pair indicated that the ratios ranged from 0.0634 (*StGGPS1*/*StGGPS4*) to 0.6052 (*StGGPS8*/*StGGPS9*), suggesting selective pressure on these genes.

To understand the evolutionary mechanisms of the potato GGPS family, we performed linear analysis using Arabidopsis and Solanaceae plants (tobacco, tomatoes, peppers, and potatoes) (Figure 3c). Four pairs of homologous genes were identified between potato and *A. thaliana*. Two homologous pairs were found between potatoes and tobacco, eight homologous genes between potatoes and tomatoes, and seven between potatoes and peppers. These homologous genes likely existed before the divergence of ancestral lineages.

### 3.4. Analysis of Cis-Acting Elements in StGGPS Gene Promoters

**Figure 4:**
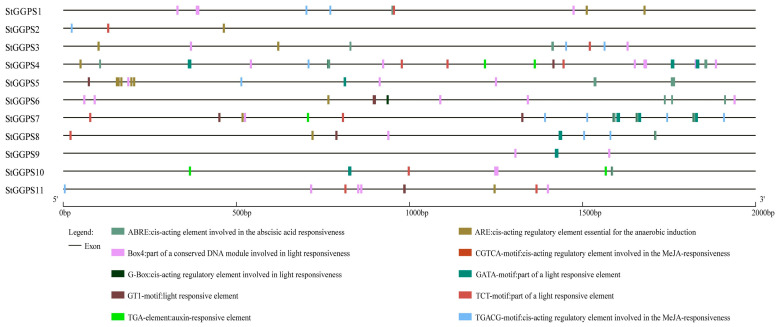
Original analysis of the cis-acting 2000 bp promoter of the *StGGPS* gene.

To explore the potential functions of *StGGPS* genes, we analyzed the 2000 bp upstream promoter sequence. We identified 10 cis-acting elements (Figure 4) associated with various physiological processes. Notably, elements such as Box4, G-box, GATA-motif, GT1-motif, and TCT-motif, which are related to light sensing, were prevalent. This suggests that the *StGGPS* gene family is involved in potato photosynthesis. Additionally, *StGGPS* genes associated with abiotic stress responses contained cis-elements such as ABRE (involved in abscisic acid response), ARE (anaerobic response), CGTCA-motif and TGACG-motif (jasmonic acid response), and TGA (auxin response). Overall, *StGGPS* genes likely play significant roles in light and abiotic stress responses in potatoes.

### 3.5. Expression Patterns of Genes in Tubers of Different Colors

**Figure 5:**
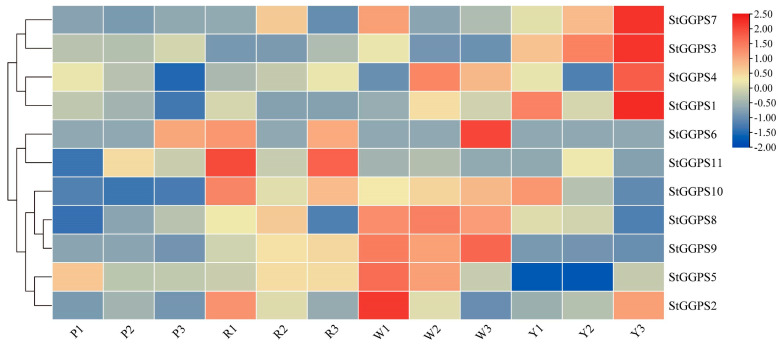
Expression of *StGGPS* gene in different colored tubers (P: purple potato; R: red potato; W: white potato; Y: yellow potato).

Given the established correlation between tuber color and carotenoid content [17], RNA-sequencing data were employed to investigate the expression patterns of *StGGPS* genes across potato tubers exhibiting four distinct colors (Figure 5). The analysis revealed differential expression of *StGGPS* genes dependent on tuber color. Specifically, *StGGPS1*, *StGGPS3*, *StGGPS4*, and *StGGPS7* were upregulated in yellow tubers, whereas *StGGPS2*, *StGGPS5*, *StGGPS8*, and *StGGPS9* showed increased expression in white tubers. Red tubers exhibited upregulation of *StGGPS6*, *StGGPS10*, and *StGGPS11*, while *StGGPS* gene expression was generally downregulated in purple tubers. These findings suggest a potentially higher carotenoid content in yellow and red tubers and a correspondingly lower carotenoid content in purple tubers. The observed downregulation of *StGGPS* genes in purple tubers likely represents a metabolic and transcriptional shift, prioritizing anthocyanin biosynthesis over carotenoid production. This adaptive reallocation of resources may serve to optimize stress tolerance or mediate ecological interactions.

### 3.6. Gene Expression Patterns in Response to Abiotic Stress

**Figure 6:**
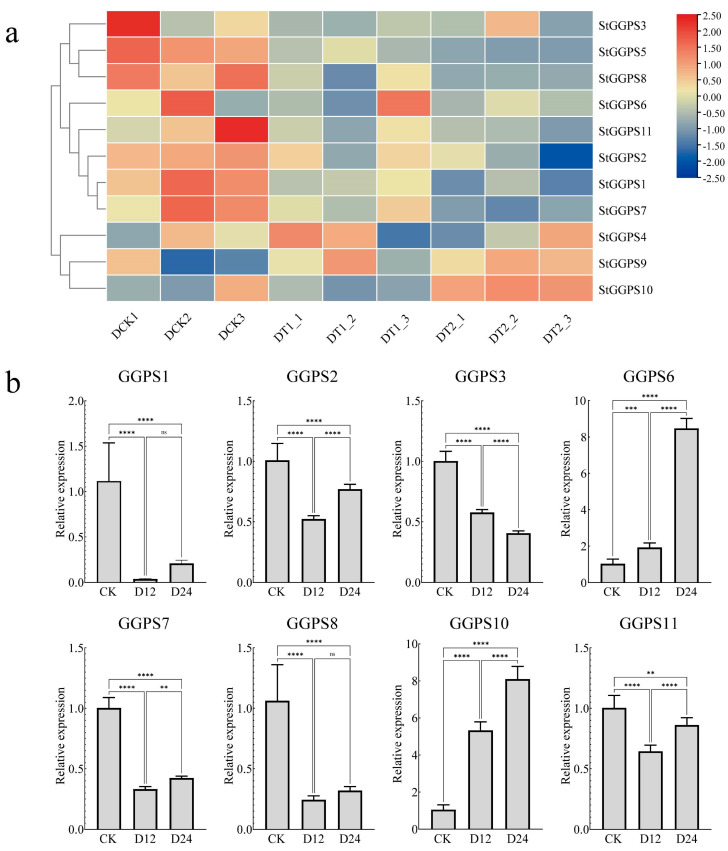
Expression of *StGGPS* gene under drought stress. (**a**): Expression of *StGGPS* under drought stress. (**b**): Relative expression of *StGGPS* under drought stress (**: *p* < 0.01, ***: *p* < 0.001, and ****: *p* < 0.0001).

**Figure 7:**
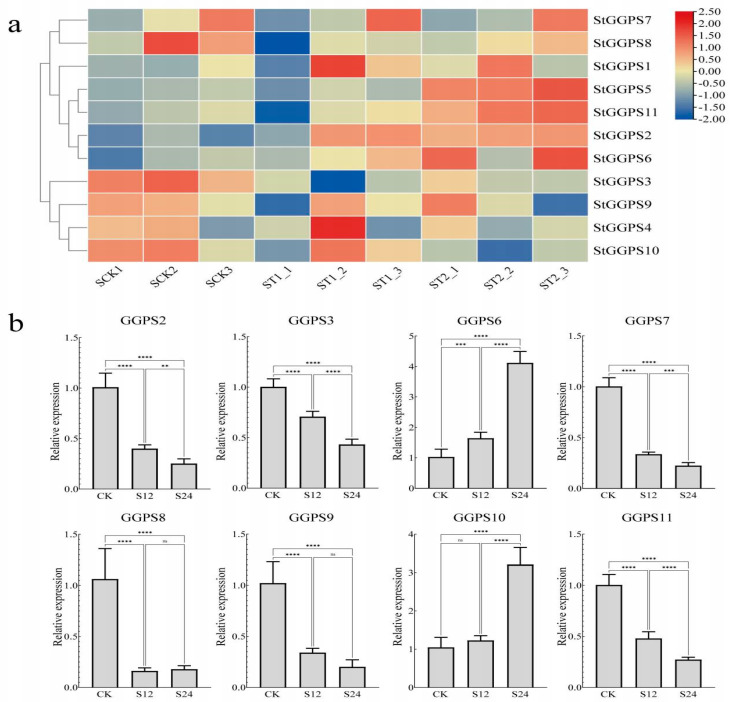
Expression of *StGGPS* gene under salt stress. (**a**): Expression of *StGGPS* under salt stress. (**b**): Relative expression of *StGGPS* under salt stress (**: *p* < 0.01, ***: *p* < 0.001, and ****: *p* < 0.0001).

Abiotic stresses, such as drought and salt stress, significantly impact potato production. To investigate the response of the *StGGPS* family to these stresses, we analyzed RNA-seq data from NCBI, validated by RT-qPCR. Under drought stress, the RNA-seq data revealed differential expression of *StGGPS* genes. Specifically, *StGGPS9* and *StGGPS10* were upregulated, whereas other *StGGPS* members were downregulated. Reverse transcription quantitative PCR validation confirmed the drought-responsive nature of *StGGPS10*, demonstrating a significant 5.2-fold increase in transcript abundance under drought stress (*p* < 0.001). This substantial induction suggests a critical role for *StGGPS10* in mediating drought resistance in potato (Figure 6). Under salt stress conditions, RNA-seq analysis revealed differential expression of multiple StGGP family members, including *StGGPS3*, *StGGPS4*, *StGGPS9*, *StGGPS10*, *StGGPS2*, *StGGPS5*, *StGGPS6*, and *StGGPS11*. Subsequent reverse transcription quantitative real-time PCR (RT-qPCR) validation confirmed the salt-responsive transcription of *StGGPS6*, which exhibited a significant 3.8-fold upregulation of transcript abundance under salt stress (*p* < 0.001) (Figure 7). Based on these findings, we hypothesize that *StGGPS6* and *StGGPS10* play important roles in the abiotic stress response of potatoes.

## 4. Discussion

The StGGPS gene family in potato comprises three phylogenetically distinct subfamilies: Group 3 (seven members), Group 2 (two members), and Group 1 (a single member). Ancient duplication events with high retention rates of gene pairs have driven the proliferation of duplicated genes in plant genomes [35]. Notably, we identified a conserved duplicate pair (*StGGPS1*/*StGGPS4*) within Group 3, suggesting this subfamily’s pivotal role in StGGPS expansion. RNA-seq analyses revealed co-expression patterns of StGGPS1 and StGGPS4 across differentially pigmented tubers, implicating their co-ordinated regulation in carotenoid biosynthesis pathways. 

Furthermore, RNA-seq and RT-qPCR analyses confirmed that *StGGPS1* was downregulated under drought stress conditions, whereas *StGGPS4* was upregulated. In contrast, under salt stress conditions, *StGGPS1* was upregulated and *StGGPS4* was downregulated. These findings suggest that *StGGPS1* and *StGGPS4* may have distinct roles in the potato plant’s response to abiotic stress. Additionally, our collinearity across species revealed that certain *StGGPS* genes are evolutionarily conserved. For instance, *StGGPS7* (PGSC0003DMG400002687) in potatoes is homologous to *NaGGPS2* (A4A49_09152) in tobacco, *SlGGPS6* (Solyc09g008920.3) in tomato, and *CaGGPS10* (T459_22463) in pepper. Additionally, *StGGPS9* (PGSC0003DMG400014369) in potatoes was homologous to *NaGGPS3* (A4A49_24015) in tobacco, *SlGGPS8* (Solyc10g005840.3) in tomatoes, and *CaGGPS11* (T459_24642) in pepper plants. These genes may have been present before the divergence of the ancestral lineages of Solanaceae and, at the same time, these orthologous genes may play similar functions and roles in different Solanaceae species.

As a key enzyme in the synthesis of terpenoids, geranyl geranyl pyrophosphate synthase (GGPS) catalyzes the production of geranyl geranyl pyrophosphate (GGPP), a crucial compound in plant photosynthesis and biotic/abiotic stress responses [15,16,36]. Previous studies have demonstrated that *NtGGPPS1* and *NtPSY1* in tobacco form a multi-enzyme complex that regulates carotenoid biosynthesis and the co-expression of genes in the carotenoid biosynthesis pathway [36]. Similarly, *ApGGPS* is the key enzyme in andrographolide synthesis and plays a role in photosynthesis, growth, and other physiological processes in plants by participating in GGPP synthesis [37]. 

Through collinearity analysis, we identified four pairs of homologous genes in potato and Arabidopsis, including *StGGPS2* (PGSC0003DMG400041508), *AtGGPS5* (AT2G18640), *AtGGPS15* (AT4G36810), *StGGPS5* (PGSC0003DMG400007081), *AtGGPS1* (AT1G17050), and *AtGGPS3* (AT1G78510). *AtGGPS15* (AT4G36810) is a key isoenzyme required for the production of most isoprene compounds involved in photosynthesis [17]. The conserved gene structures and motifs between *StGGPS2* and *AtGGPS15* suggest functional conservation, potentially underpinning their shared roles in photosynthetic processes of potato. In addition, *AtGGPS1* (AT1G17050) and *AtGGPS3* (AT1G78510) are paralogous genes involved in the synthesis of [20] the REDOX cofactor plastoquinine-9 in photosynthesis [38], a key antioxidant in plant leaves that plays a critical role in photoprotection [39]. Higher plastoquinin-9 activity in potatoes could enhance resistance to the effects of salt stress on photosynthesis. *StGGPS5* is homologous to *AtGGPS1* (AT1G17050) and *AtGGPS3* (AT1G78510), and RNA-seq data show significant upregulation of *StGGPS5* under salt stress; we propose that *StGGPS5* may be closely related to plastoquinone-9 synthesis in potatoes and participate in their response to salt stress, thereby mitigating the adverse effects of salt on potato growth and development.

Future research should integrate metabolomic profiling with transgenic approaches, such as the overexpression of StMYB1, to rigorously validate the proposed metabolic trade-off between carotenoid and anthocyanin biosynthesis in potato tubers. Further elucidation of the transcriptional cross-regulation and resource allocation mechanisms governing these pathways is crucial for a more complete understanding of their competitive interaction. Furthermore, characterizing the ecological advantages conferred by anthocyanin dominance may inform strategies aimed at engineering stress-resilient cultivars with tailored nutritional profiles. This will allow for a more informed balancing of pigmentation and stress adaptation traits for the improvement of agricultural outcomes.

## 5. Conclusions

Eleven *StGGPS* family genes were identified and classified into three subfamilies based on gene structure and conserved motif analysis. Bioinformatics analysis and gene expression verification revealed that these genes contain numerous photo-responsive and cis-acting elements related to abiotic stress responses. Combined with transcriptome data and q-PCR verification, our findings suggest that *StGGPS* family members play a significant role in helping potatoes resist damage caused by abiotic stress, thereby supporting the plant’s growth and development under adverse conditions.

## Figures and Tables

**Table 1:** Analysis of potato GGPS gene family members and physicochemical properties.

Gene ID	Gene Name	Chromosome Localization				Amino Acid Length (aa)	Molecular Weight (Da)	Theoretical Isoelectric Point	Subcellular Localization
PGSC0003DMG400047044	*StGGPS1*	chr02	40939136	40940224	+	362	39,321	6.35	Chloroplast
PGSC0003DMG400041508	*StGGPS2*	chr02	40941168	40942286	+	372	41,120.1	8.38	Mitochondrion
PGSC0003DMG400043267	*StGGPS3*	chr02	40945073	40946191	+	372	40,731.9	7.92	Chloroplast
PGSC0003DMG400027856	*StGGPS4*	chr04	69344146	69345467	−	375	40,657.5	4.99	Chloroplast
PGSC0003DMG400007081	*StGGPS5*	chr07	52190753	52195395	−	398	43,490.4	6.01	Chloroplast
PGSC0003DMG400022214	*StGGPS6*	chr07	54896132	54897257	−	294	32,784.6	7.01	Cytoskeleton
PGSC0003DMG400002687	*StGGPS7*	chr09	4028005	4032971	+	334	36,484.6	5.26	Chloroplast
PGSC0003DMG400008690	*StGGPS8*	chr10	854194	861002	+	342	39,650.3	6.12	Cytoplasm
PGSC0003DMG400014369	*StGGPS9*	chr10	871271	879652	+	306	35,198.2	6.77	Nucleus
PGSC0003DMG400015673	*StGGPS10*	chr11	1682159	1683721	−	365	39,976.7	6.85	Chloroplast
PGSC0003DMG400029788	*StGGPS11*	chr12	10169940	10174778	−	342	39,296.9	4.87	Cytoplasm

## Data Availability

Data are contained within the article and Supplementary Materials.

## Supplementary Materials

The following supporting information can be downloaded at: https://www.mdpi.com/article/10.3390/genes16060646/s1, Figure S1: High resolution images used in this article; Table S1: The primer sequences used in this study.
