# Supplementary figures and images for: Genome-Wide Identification of the Potato GGPS Gene Family and Analysis of Its Response to Abiotic Stress
Source: Genes (Basel). 2025 May 28;16(6):646. doi: 10.3390/genes16060646 (PMC12192103; doi:10.3390/genes16060646)

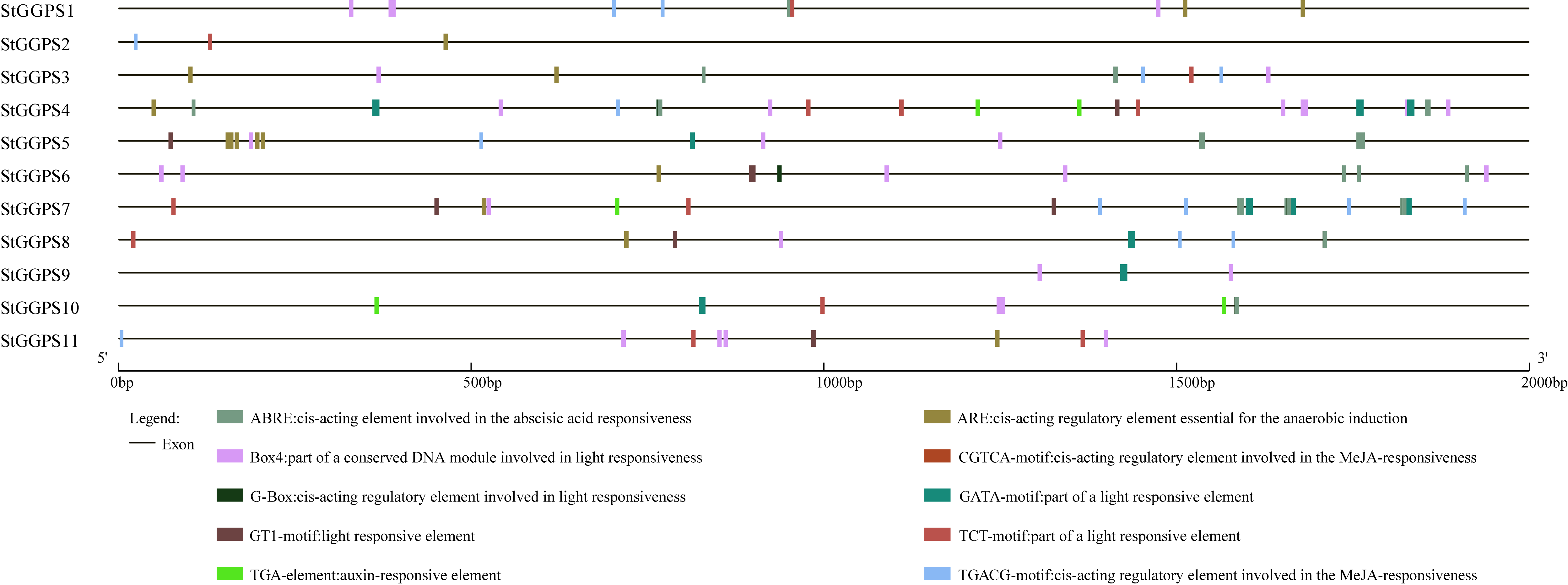

Supplement: Supplementary file 1 [file genes-16-00646-s001.zip › Raw data, charts/Chart attachment/Fig.4.tif]

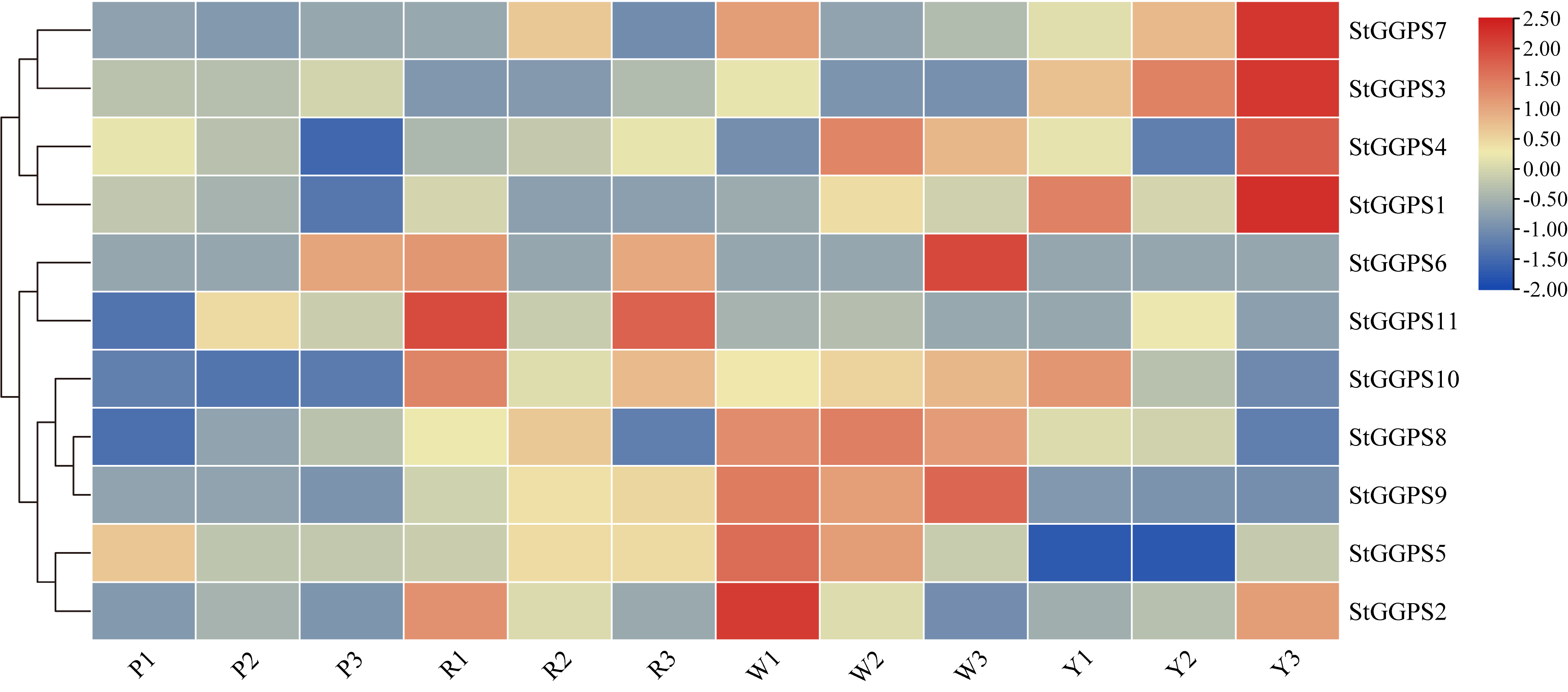

Supplement: Supplementary file 1 [file genes-16-00646-s001.zip › Raw data, charts/Chart attachment/Fig.5.tif]

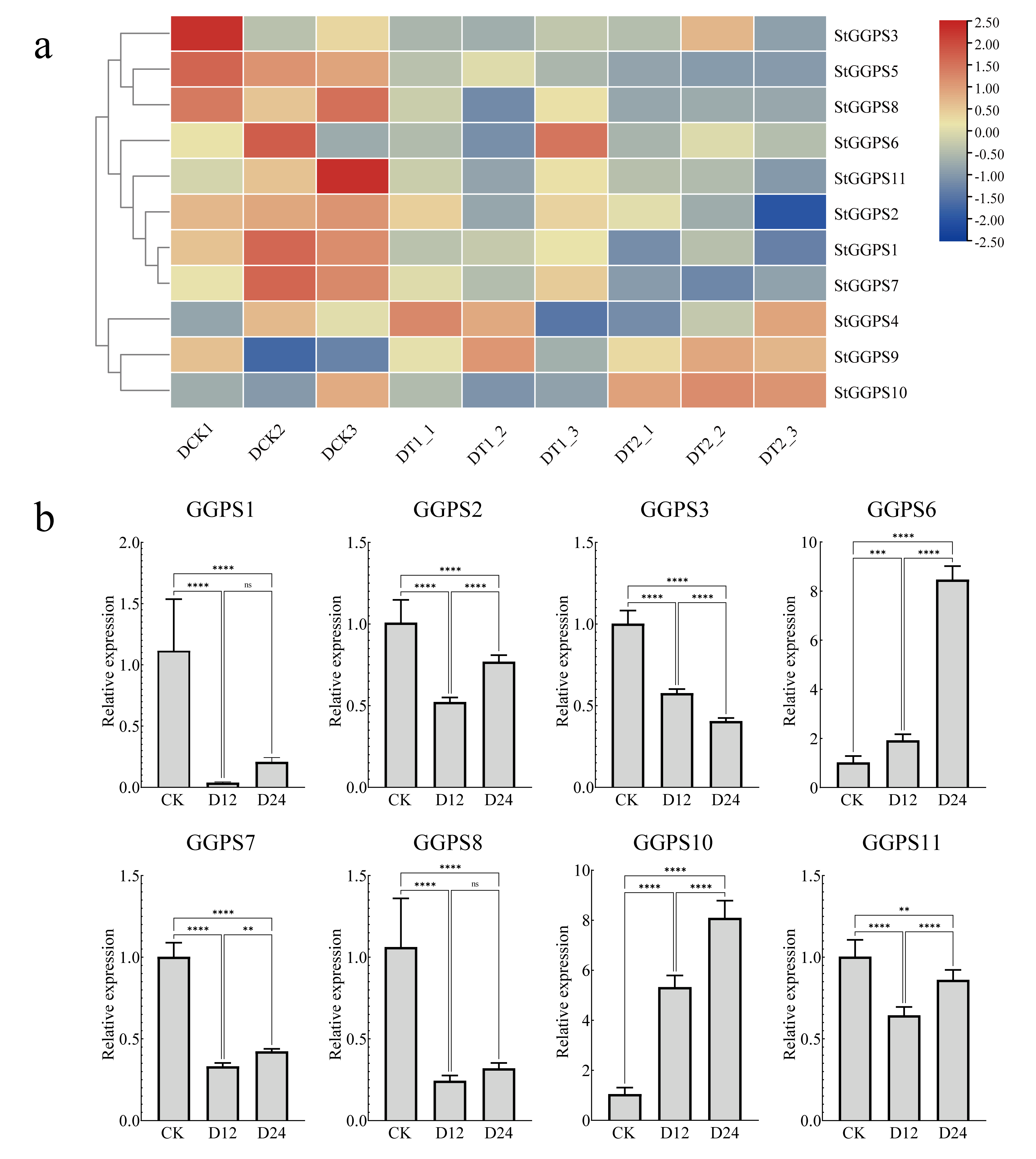

Supplement: Supplementary file 1 [file genes-16-00646-s001.zip › Raw data, charts/Chart attachment/Fig.6.tif]

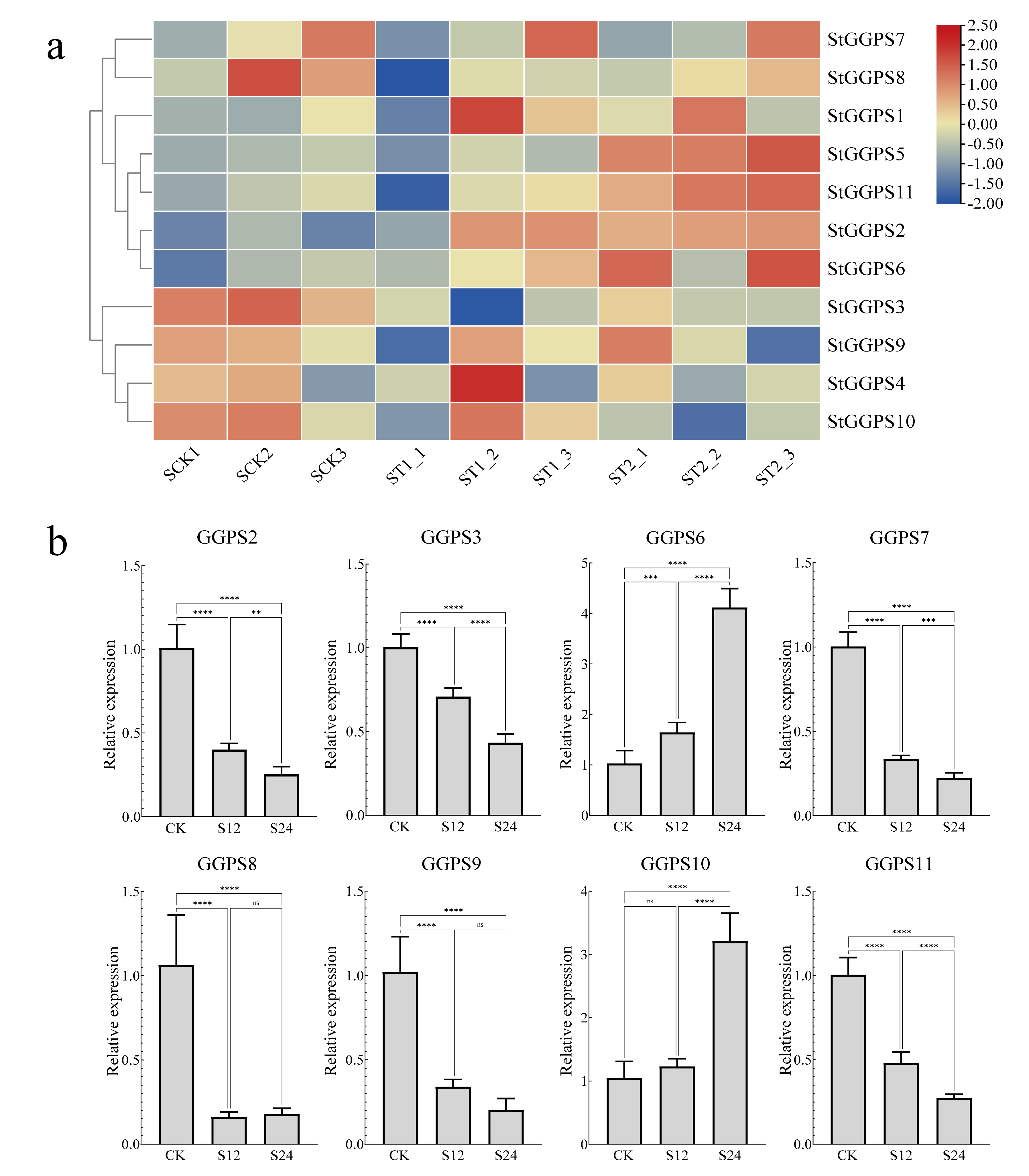

Supplement: Supplementary file 1 [file genes-16-00646-s001.zip › Raw data, charts/Chart attachment/Fig.7.tif]
